# Optimal positive end-expiratory pressure in mechanically ventilated patients: a clinical study

**DOI:** 10.1186/cc9615

**Published:** 2011-03-11

**Authors:** A Sundaresan, JG Chase, CE Hann, GM Shaw

**Affiliations:** 1 University of Canterbury, Christchurch, New Zealand; 2 Christchurch Hospital, Christchurch, New Zealand

## Introduction

The optimal level of positive end-expiratory pressure (PEEP) is still widely debated in treating acute respiratory distress syndrome (ARDS) patients. Current methods of selecting PEEP only provide a range of values and do not provide unique patient-specific solutions. Model-based methods offer a novel way of using non-invasive pressure-volume (PV) measurements to estimate patient recruitability. This paper examines the clinical viability of such models in pilot clinical trials to assist therapy, optimise patient-specific PEEP, and assess the disease state and response over time.

## Methods

Ten patients with acute lung injury or ARDS underwent incremental PEEP recruitment manoeuvres. PV data were measured in increments of 5 cmH_2_O and fitted to the recruitment model using volume-controlled ventilation. Inspiratory and expiratory breath holds were performed to measure airway resistance and auto-PEEP. Three model-based metrics are used to optimise PEEP based on threshold opening pressures (TOP), threshold closing pressures (TCP) and net recruitment. ARDS status was assessed by model parameters capturing recruitment and compliance. Two patients underwent multiple recruitment manoeuvres over time and four model metrics reflected and tracked the state or their ARDS.

## Results

Median model fitting error across all patients for inflation and deflation was 2.8% and 1.02%, respectively, with all patients experiencing auto-PEEP. In all three metrics cases, model-based optimal PEEP was higher than clinically selected PEEP. Ranges for optimal PEEP were (5, 27), (10, 25) and (10, 30) cmH_2_O for TOP, TCP and net recruitment metrics, respectively. Disease-tracking metrics corresponded with the physiological status of two patients, indicating the potential for tracking disease state. In particular, monitoring TOP, standard deviation, TOP gradient and TCP gradient reflected compliance and recruitability changes as a function of time. Normalised SD reflected compliance changes in an exponential manner with the equation 72.6 × exp^-0.0664 × SD^, indicating the model's utility in evaluating true lung linear compliance.

## Conclusions

For ARDS patients, the model-based method presented in this paper provides a unique, non-invasive method to select optimal patient-specific PEEP. In addition, the model has the capability to assess disease state over time and monitor patient status.

